# A 7-Years Active Pharmacovigilance Study of Adverse Drug Reactions Causing Children Admission to a Pediatric Emergency Department in Sicily

**DOI:** 10.3389/fphar.2020.01090

**Published:** 2020-07-17

**Authors:** Chiara Nasso, Anna Mecchio, Michelangelo Rottura, Mariella Valenzise, Francesca Menniti-Ippolito, Paola Maria Cutroneo, Violetta Squadrito, Francesco Squadrito, Giovanni Pallio, Natasha Irrera, Vincenzo Arcoraci, Domenica Altavilla

**Affiliations:** ^1^Department of Clinical and Experimental Medicine, University of Messina, Messina, Italy; ^2^Department of Biomedical and Dental Sciences and Morphofunctional Imaging, University of Messina, Messina, Italy; ^3^Department of Human Pathology in Adulthood and Childhood “Gaetano Barresi”, University of Messina, Messina, Italy; ^4^National Centre for Drug Research and Evaluation, National Institute of Health, Rome, Italy; ^5^Sicilian Regional Pharmacovigilance Center, Clinical Pharmacology Unit, University Hospital of Messina, Messina, Italy

**Keywords:** adverse drug events, adverse drug reactions, children, pediatric emergency department, pharmacovigilance, safety

## Abstract

Children represent one of the most susceptible groups to adverse drug reactions (ADRs), as a consequence of physiological growth and maturation of different organ systems. The aim of this study was to characterize the frequency, preventability and seriousness of ADRs recorded in the Pediatric Emergency Department (ED) of the University hospital of Messina, in Sicily. All the suspected adverse reactions to drugs and vaccines collected from 2012 to 2018 were selected and then analyzed. Only adverse drug reactions (ADRs) with a probable or possible causality assessment were included, according to the Naranjo Algorithm and the World Health Organization criteria; the preventability assessment using Schumock and Thornton criteria was also carried out. The Medical Dictionary for Regulatory Activities (MedDRA) was used to group ADRs. Of 75,935 admissions to the Pediatric ED, 120 were due to suspected ADRs. The rate of hospital admission due to ADRs (75.8%) was significantly greater than that of patients without ADRs (11.9%). Among pediatric patients with ADRs the median (Q1–Q3) age was 29.5 (12–73.25) months. Most of ADRs were observed in infants and children (43.3% and 41.7%, respectively vs adolescents, 15%). In addition, in children with ADRs, females [41 (14–105)] were older than males [23 (11–45)] (p=0.044). Most adverse reactions were serious (75.8%) and 20.8% were preventable or probably preventable; however, the majority of serious ADRs (93.4%) resulted without sequelae. The reactions were found to be as probable (54.2%) or possible (45.8%). Vaccines (n=63), antibacterials (n=31) and anti-inflammatory medicines (n=14) were the most frequently drugs involved. Organ toxicity mapping due to vaccines was general disorders and administration site conditions (65.1%), nervous disorders (50.2%), cutaneous disorders (35%), followed by gastrointestinal disorders (20.6%). Cutaneous disorders (76%) gastrointestinal (20.7%), general (15.5%), and nervous disorders (8.6%) were the organ toxicity mapping due to drugs. Active pharmacovigilance has an essential role in supporting the development of strategies aimed at intervention to reduce admissions due to ADRs. Our data suggest that ADRs represent the first cause of hospitalization to the Pediatric Emergency Department. Furthermore, according to the literature, vaccines and antibiotics are the most frequent cause of adverse drug reactions in children.

## Introduction

Post-marketing surveillance in children is a useful source to monitor the safety of the drugs used in pediatrics. The World Health Organization (WHO) defines the adverse drug reaction (ADR) as a “response to a drug that is noxious and unintended and which occurs at doses normally used in man” (Wong et al., 2019).

The ADRs represent a public health problem, particularly in pediatric patients (Andrade et al., 2017a) and often are the cause of emergency department (ED) admission. In the last years, an increased number of ED visits of pediatric population has been observed, thus causing an overcrowding in the hospital. Several factors may influence the ED visits such as age, gender and familiar socioeconomic characteristics (Riva et al., 2018).

ED visits represent a significant source of information in terms of frequency, preventability, and seriousness of ADRs (Lombardi et al., 2018). Moreover, 1/10 of in-hospital children have an ADR, 12% of them are serious (Clavenna and Bonati, 2009) and 2.9% of hospitalizations were due to ADRs (Smyth et al., 2012).

Several reports suggest that the rate of ADR-related ED admissions in children is lower (5%) than in adults (25%) (Lombardi et al., 2018). An active monitoring system of ADR showed that neonates (< 1 years) have an higher risk of ADR appearance than children (7–14 years) (Menniti-lppolito et al., 2000). Antibacterial for systemic use were reported as the drugs with the highest frequency of ADRs (Carnovale et al., 2014; Ferrajolo et al., 2014; Lombardi et al., 2018)

In the recent years, the number of vaccine-hesitant patients as well as the movements against vaccination grew up (Lombardi et al., 2019). Consequently, immunization coverage of children sharply decreased (Signorelli et al., 2017). However, the number of vaccinated children is rising again following the introduction of Italian National Health Service intervention advise (Law n. 119, July 2017). During 2018, 17.6% immunization related adverse events (AEFI) of a total of 7,315 were defined as serious, according to the Italian Drug Agency (AIFA) (Agenzia Italiana del Farmaco, 2018).

Indeed, the physiological characteristics of children are different from those of adults, because of immature and not yet fully developed systems, in particular renal and hepatic enzyme systems (Anderson, 2002). Therefore, children are often more vulnerable and differently respond to drugs (Andrade et al., 2017b). The most affected organs were skin and subcutaneous tissue followed by the gastrointestinal tract (Priyadharsini et al., 2011).

The reported rate of adverse reactions severity and avoidance, are highly variable. Indeed, a systematic review shows that the severity rates vary from 0% to 66.7% while avoidance from 7% to 98% (Smyth et al., 2012).

Randomized controlled trials (RCTs) are not useful to detect ADRs in the pediatric population, especially in neonates and infants (Nor Aripin et al., 2012). These studies do not consider drug toxicity, the recommended dose and warning issues (Star et al., 2019).

Thus, spontaneous ADRs reporting could improve information on drugs safety and on seriousness of ADRs. In this regard, the role of pharmacovigilance is of paramount importance to prevent ADRs in children and to limit ED admission.

In the light of the foregoing considerations, the main aim of the present study was to analyze the frequency, preventability and seriousness of ADRs as well as hospitalization due to ADRs, retrieved from the Pediatric Emergency Department of the University hospital of Messina, in a framework of seven years. Secondary end-points were to characterize patients with ADRs according to seriousness and to evaluate the therapeutic groups related to ADRs.

## Materials and Methods

### Setting

The study was carried out between the years 2012 and 2018, in the Pediatric Emergency Department (ED) of the University Hospital “G.Martino” of Messina, (Sicily, Italy). In this prospective study, we considered ADRs data that were collected, prospectively, as part of a pharmacovigilance multi-center project on drug and vaccine safety in the pediatric populations, coordinated by the National Institute of Healthcare in Italy. In the University Hospital of Messina there is a specific pediatric ED insuring first aid assistance to people until 16 years within a population of about 250,000 citizens. Information were retrieved from patients visited in the ED by the monitors, who have received specific training on pharmacovigilance in the pediatric setting. The monitors also followed up the patients during hospitalization to verify the outcome.

### Ethical Approval and Consent to Participate

This pharmacovigilance project was approved by the Ethics Committee of Messina University Hospital (n° prot E22/12; Coordinator Centre) according to the legal requirements concerning observational studies. Patient’s consent to participate is not requested for this kind of study. In accordance with specific activities of the Emergency Department, generally of urgent nature, the collection of informed consent is not compatible with the possibility of guaranteeing the conduct of normal clinical practice.

### Data Collection

In the study period, the monitors prospectively screened all admissions to the pediatric ED.

ADRs were identified by reviewing patients’ records, and interviewing their parents to clarify symptoms and timing of events, when possible.

A research teams of pharmacologists, operating in the Sicilian Pharmacovigilance Centre of the University Hospital of Messina, ED physicians and monitors reviewed all identified cases of ADRs. In particularly, this group analyzed each case of suspected ADR, to evaluate the relation between drug administration and ADR onset using the Naranjo algorithm (Naranjo et al., 1981; Lombardi et al., 2018). Preventability of ADRs was also evaluated, using the modified Schumock and Thornton criteria (Schumock and Thornton, 1992).

The following information were recorded for each patient, following experiencing an ADR: sociodemographic characteristics (age, gender, clinical history, and allergies), suspected and concomitant drugs (dosage, route of administration, duration, and therapeutic indication), characteristics of ADRs onset, seriousness, hospital length of stay (LOS) and outcomes. Data were collected in a standardized format, developed *ad hoc*. ADRs were categorized as acute (occurring within 24 h) or latent (occurring after 24 h). Onset was defined as the time from the start of drug administration to the beginning of a reaction. According to the World Health Organization (WHO) criteria, ADRs were classified as serious, when are fatal, life-threatening, requiring hospitalization of the patient, or causing serious/permanent disability.

Drugs were classified according to Anatomical Therapeutic Chemical Classification (ATC). ADRs were codified as detailed by the Medical Dictionary for Regulatory activities (MedDRA^®^) and grouped according to the System Organ Classes (SOCs) classification and preferred terms (PTs). ADRs were stratified by group age based on the International Conference on Harmonization guideline on Clinical Investigation of Medicinal Products in the Pediatric Population (European Medicines Agency, 2017).

The rate of ED admissions due to ADR was evaluated as the ratio between the number of patients admitted for ADRs and the total number of ED admissions per 1,000 patients. The rate of hospitalization due to ADRs was calculated as the ratio between the number of patients’ hospitalization for ADRs and the total patients’ hospitalization.

In accordance with the Italian legal requirements, all collected ADRs were also reported into the Italian National Pharmacovigilance Network coordinated by the Italian Medicines Agency (AIFA).

### Statistical Analysis

Descriptive statistical analyses were performed to evaluate basal demographic, clinical characteristics, and drug-related variables of patients with ADRs. Suspected drugs, seriousness, outcome, occurring ADR, latency time, and type of ADR were also evaluated. All results were expressed as medians with interquartile ranges (Q1–Q3), for continuous variables, and absolute and percentage frequencies for categorical variables. The Pearson’s chi-squared test was used to compare categorical variables (gender, age group, causality, preventability, outcomes, and therapeutic groups) according to seriousness, onset and preventability of ADRs. The Mann–Whitney U test was performed to compare continuous variables (age) according to gender. A p-value <0.05 was considered statistically significant. Statistical analysis was performed with SPSS version 23.0 (IBM Corp., SPSS Statistics, Armonk, NY, USA).

## Results

A total of 75,935 children were admitted to the pediatric ED throughout the study period, and 9,111 (12.0%) of them resulted in hospitalization. Overall, 120 admissions were caused by an ADR (1.6 ADRs/1,000 admissions) and 91 of them resulted in hospitalization. The rate of hospitalization due to ADRs was 1.0% (91/9,111). The rate of hospitalization in children with ADRs (91/120; 75.8%) was significantly greater than in patients without ADRs (9,020/75,815; 11.9%; p <0.001). The LOS of patients hospitalized because of ADRs was 3 (0–6) days.

The median age of pediatric patients was 29.5 (12.0–73.2) months and 51.7% of them were females. Most ADRs were observed in infants and children (43.3% and 41.7%, respectively). In addition, females with ADRs were older than males [median age: 41 (14–105) vs 23 (11–45); (p=0,044)]. Most patients had at least one serious ADR (n=91, 75.8%).

Gender, age groups, causality, preventability, outcomes, and therapeutic groups, according to seriousness of ADR, are reported in **Table 1**. No significant differences were observed between patients reporting serious or not serious ADRs. One ADR resulted in a significant disability (preventable hepatotoxicity related to paracetamol) in a 2-month-old infant after 3 days of drug treatment. No uncertain ADRs were found in our study.

**Table 1:** Seriousness of ADRs.

	Total N 120 (%)	Serious N 91 (%)	Non–serious N 29 (%)	P value
**Gender**				
Female	62 (51.7%)	45 (49.4%)	17 (51.7%)	0.389
Male	58 (48.3%)	46 (50.6%)	12 (41.4%)	
**Age groups**				
Infants	52 (43.3%)	44 (48.3%)	8 (27.6%)	0.088
Children	50 (41.7%)	33 (36.3%)	17 (58.6%)	
Adolescents	18 (15.0%)	14 (15.4%)	4 (13.8%)	
**Causality**				
Possible	55 (45.8%)	45 (49.4%)	10 (34.5%)	0.159
Probable	65 (54.2%)	46 (50.6%)	19 (65.5%)	
**Preventability**				
Preventable/probably preventable	25 (20.8%)	22 (24.2%)	3 (10.3%)	0.110
Not preventable	95 (80.2%)	69 (75.8%)	26 (89.7%)	
**Outcomes**				
Improvement	8 (6.7%)	5 (5.5%)	3 (10.3%)	0.569
Recovery without consequences	111 (92.5%)	85 (93.4%)	26 (89.7%)	
Resulted in persistent of significant disability or incapacity	1 (0.8%)	1 (1.1%)	–	
**Therapeutic Group**				
Vaccine	63 (52.5%)	47 (51.6%)	16 (55.2%)	0.741
Drug	57 (47.5%)	44 (48.4%)	13 (44.8%)	

ADRs, adverse drug reactions.

The percentage of preventable/probable preventable ADRs significantly decreased with the age of the groups (adolescents 44,4% vs children 26,0% vs infants 7,7%; p=0.002).

Vaccine related preventable/probable preventable ADRs (12.7%) were significantly lower that drugs related ones (29.8%); (p = 0.021).

Gender, causality, seriousness, and outcomes did not show statistically significant differences according to preventability.

**Figure 1:**
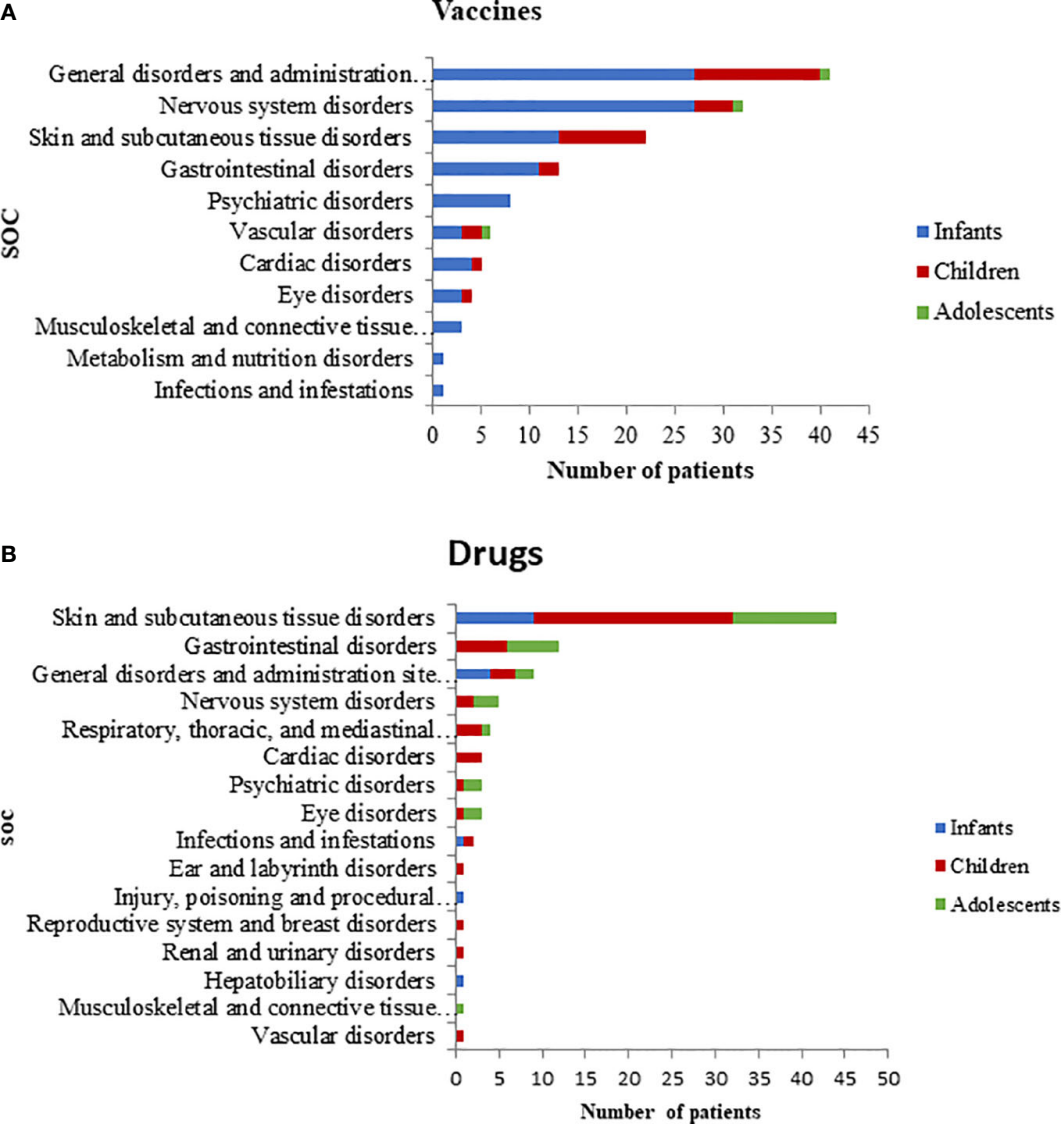
System organ class for vaccine **(A)** and drug **(B)** according to age group.

Most ADR reports (38/63) had only one vaccine as suspected drug; 22 out of 63 had two vaccines, and 3 out of 63 had three vaccines. Viral, bacterial, and combined vaccines contributed to 17.5%, 25.4% and 57.1% of the reports, respectively. Of these, the 17.5% was only pre-combined vaccines; the 33.3% was pre-combined vaccines associated with bacterial vaccines; the 4.8% involved pre-combined vaccines with bacterial and viral vaccines and the 1.6% was an association of bacterial vaccine with a viral vaccine. The therapeutic groups are reported in **Table 2**, according to seriousness of ADRs. Patients with at least one ADR related to the administration of an antibacterial for systemic use were mostly children (54.8%) followed by infants (22.6%) and adolescents (22.6%). Furthermore, ADRs regarding anti-inflammatory and anti-rheumatic products were mainly observed between adolescents and children (50.0% and 42.9%, respectively) followed by infants (7.1%). The majority of ADRs induced by vaccines, anti-inflammatory and anti-rheumatic products were acute reactions, occurred within 24 h after drug exposure. Conversely, antibacterials for systemic use, analgesics and drugs for obstructive airway diseases were mostly latent reactions. ADRs onset related to therapeutics groups are listed in **Table 3**. The SOCs involved for drugs and vaccines were analyzed separately, according to children’s age groups (**Figures 1A, B**). Within 63 ADRs reports of vaccines, there were 136 SOCs. The most commonly reported SOCs were general disorders and administration site conditions (65.1%) while the most frequently observed reactions were fever, gait abnormality, asthenia and injection site pain. Other common SOCs were nervous (50.2%), cutaneous (35%) and gastrointestinal disorders (20.6%). All of these SOCs were mostly observed in infants.

**Table 2:** Therapeutic groups (ATC 2nd Level) associated with adverse drug reactions and stratified by seriousness.

	ATC Level 2	Serious ADRsN (%)	Non-seriousADRsN (%)	TotalADRs^a^N (%)
**J07**	Vaccines	47 (51.6)	16 (55.2)	63 (52.5)
**J01**	Antibacterials for systemic use	26 (28.6)	5 (17.2)	31 (25.8)
**M01**	Antinflammatory and antirheumatic products	11 (12.1)	3 (10.3)	14 (11.7)
**N02**	Analgesics	5 (5.5)	—	5 (4.2)
**R03**	Drugs for obstructive airway diseases	2 (2.2)	2 (6.9)	4 (3.3)
**N03**	Antiepileptics	4 (4.4)	—	4 (3.3)
**H02**	Corticosteroids for systemic use	2 (2.2)	—	2 (1.7)
**S01**	Ophthalmologicals	0 (-)	3 (10.3)	3 (2.5)
**R05**	Antitussives	1 (1.1)	1 (3.4)	2 (1.7)
**R06**	Antihistamines for systemic use	1 (1.1)	—	1 (0.8)
**N05**	Psycholeptics	1 (1.1)	—	1 (0.8)
**C09**	Agents acting on the renin-angiotensin system	1 (1.1)	—	1 (0.8)
**L01**	Antineoplastic and immunomodulating agents	1 (1.1)	—	1 (0.8)
	**Total**	91 (100)	29 (100)	120 (100)

ADRs, adverse drug reactions; ATC, Anatomical Therapeutic Chemical.

^a^The total number of ADRs reported in the table does not coincide with the total of reports because in each individual reporting form may be indicated one or more suspected active substances that are possibly responsible for the reported ADRs.

**Table 3:** Number of adverse drug reactions for each therapeutic group (ATC 2nd Level) stratified by onset.

	ATC Level 2	Total ADR	Acute	Latent
**J07**	Vaccines	92 (%)	63 (68.5)	29 (31.5)
**J01**	Antibacterials for systemic use	32 (%)	9 (28.1)	23 (71.9)
**M01**	Antinflammatory and antirheumatic products	15 (%)	10 (66.7)	5 (33.3)
**N02**	Analgesics	5 (%)	2 (40.0)	3 (60.0)
**R03**	Drugs for obstructive airway diseases	4 (%)	1 (25.0)	3 (75.0)
**N03**	Antiepileptics	4 (%)	—	4 —
**H02**	Corticosteroids for systemic use	2 (%)	1 (50.0)	1 (50.0)
**S01**	Ophthalmologicals	3 (%)	2 (66.7)	1 (33.3)
**R05**	Antitussives	2 (%)	2 —	—
**R06**	Antihistamines for systemic use	1 (%)	—	1 —
**C09**	Agents acting on the renin-angiotensin system	1 (%)	1 —	—
**N05**	Psycholeptics	1 (%)	1 —	—
**L01**	Antineoplastic and immunomodulating agents	1 (%)	—	1 —

ADR, adverse drug reactions; ATC, Anatomical Therapeutic Chemical.

Drugs-related ADRs were observed in 57 reports, with 92 reported SOCs, mostly associated to children and adolescents (81% and 50%, respectively). Unlike vaccines, cutaneous disorders (76%) were the SOCs most frequently involved by drugs, followed by gastrointestinal (20.7%), general (15.5%), and nervous disorders (8.6%).

Overall, the 120 reports associated with vaccines and drugs included 226 ADR types (mean 1.9 ADRs per report), mainly pyrexia (n=44, 70%), vomiting (n=8, 12.7%), loss of consciousness (n=8, 12.3%), cyanosis (n=7, 11.1%), erythema (n=6, 9.5%), tremor (n=6, 9.5%), and gait abnormality (n=6, 9.5%). Furthermore, urticaria (n=9, 15.5%), erythema (n=6, 12.3%), pruritus (n=4, 7%), maculopapular rash (n=3, 5.2%), angioedema (n=2, 3.4%) and dyspnea (n=2, 3.4%) were the ADRs most frequently related with drugs. In **Tables 4** and **5**, ADRs are reported according to different vaccines or drugs.

**Table 4:** ADRs reports according to single vaccine involved.

Vaccine (No. Reports n=63)	Most common preferred terms (No. ADRs ≥ 2)
**J07AL02**—Pneumococcus, purified polysaccharides antigen conjugated **(n=35)**	pyrexia (16), hypotonia (4), loss of consciousness (4) vomiting (4), irritability (3), gait abnormality (3), cyanosis (3), febrile convulsion (3), diarrhea (3),asthenia (2), urticaria (3) tremor (3), muscle rigidity (2), erythema (2), injection site pain (2), infantile spasms (2), eating disorders (2),erythema (2), pallor (2), gaze (2), sleepiness(2)
**J07CA09**—Diphtheria-hemophilus influenzae B-pertussis-poliomyelitis-tetanus-hepatitis B **(n=30)**	pyrexia (16), febrile convulsion (3), tremor (3), cyanosis (4), vomiting (4), loss of consciousness (4), hypotonia (3), gait abnormal (3), oculogyration (2), irritability (2), diarrhea (3), muscle rigidity (2), asthenia (2), erythema (2),pallor (2), sleepiness (2),
**J07CA02**—Diphtheria-pertussis-poliomyelitis-tetanus (**n=8**)	pyrexia (3), erythema (2)
**J07AH09**—Meningococcus B, multicomponent vaccine **(n=7)**	pyrexia (4), seizures (2)
**J07BH01**—Rota virus, live attenuated **(n=7)**	pyrexia (3), intussuspection (3), diarrhea (2)
**J07BD52**—Measles, combinations with mumps and rubella, live attenuated **(n=6)**	pyrexia (2)

ADRs, adverse drug reactions.

**Table 5:** ADRs reports according to single drug involved.

Drug (No. Reports n=57)	Most common preferred terms (No. ADRs ≥ 2)
**J01CR02**—Amoxicillin and beta-lactamase inhibitor **(n=19)**	erythema (4), exanthema (2), urticarial (5), pruritus (4)
**N02BE01**—Paracetamol **(n=12)**	urticaria (2)
**M01AE01**—Ibuprofen **(n=7)**	erythema (2), exanthema macular (2)
**J01CA04—**Amoxicillin **(n=6)**	exanthema maculo papulare (3)
**M01AE03**—Ketoprofen **(n=5)**	angioedema (2)
**J01DD04**—Ceftriaxone **(n=3)**	eating disorders (2)
**J01DD08**—Cefxime **(n=3)**	urticaria (2)

ADRs, adverse drug reactions.

## Discussion

This prospective study was carried out to identify and characterize ADRs reported in a pediatric ED in Southern Italy throughout a 7-year period. More precisely, the key goal of the present study was to investigate the frequency, preventability and seriousness of ADRs as well as hospitalization due to ADRs, while the secondary end-points were to identify patients with ADRs in agreement to seriousness and to evaluate the therapeutic groups related to ADRs. The key findings of our study may be summarized in a 1) more reduced ADR induced ED admissions than previously shown in the literature; 2) an higher hospitalization rate in children with ADRs; 3) an age dependency of preventable/probable preventable ADRs; 4) a preventable ADRs pattern higher in drugs than in vaccines; 5) the highest number of ADRs for the therapeutic group of vaccines; 6) the evidence that skin and subcutaneous tissue were the most affected organs by ADRs.

Indeed, this is the first study that has been carried out in Sicily and therefore it may serve as benchmark for future research and it may offer an important piece of evidence for healthcare stakeholders and government decision makers to implement the needed actions to curb the burden of the ADRs in the pediatric age. The observed rate of ED admissions related to ADRs (1.6/1000) was lower than the rate previously reported in other studies (Lombardi et al., 2018). On the other hand, the rate of hospitalization due to ADRs in our pediatric population (1.0%) agrees with the ADRs related hospitalization previously reported (Smyth et al., 2012).

Interestingly, in the present study, over 75.8% of children who reported ADRs were hospitalized and this frequency was higher than that of hospitalized patients without ADRs (11,9%). An overuse of ED pediatric admissions which is related to an overuse of healthcare resources was previously observed (Riva et al., 2018). Even in our study, the difference in the frequency among the pediatric population might be due to an overuse of the accesses in the pediatric ED compared to the real need: this is probably due to the parental attitude of easily considering pediatric ED admission. Furthermore, the reduction of emergency medical service in Italy may have led to an increase of admissions in pediatric ED also for patients that do not require a visit. On the contrary, the admissions due to an ADR might be likely related to severe symptoms, thus increasing the probability of hospitalization.

In agreement with Menniti Ippolito et al., infants and children had a higher prevalence of ADRs (Menniti-lppolito et al., 2000). Children probably show a higher frequency of infections due to school attendance and, at the same time, they have a greater difficulty communicating their symptoms. As a consequence, some events are not recognized or perhaps erroneously interpreted as restlessness or sleepiness (Andrade et al., 2017b). Nevertheless, children have a lower risk of urgent access respect to infants. On the contrary, infants generally need an urgent access due to the seriousness of disease (Riva et al., 2018). In our study, no difference by age was observed between patients affected by serious or not serious ADRs. However, accordingly to previous study, our results highlight a greater frequency of serious ADRs in infants respect to children or adolescent, even if the statistical significance is not reached probably because of the low sample size. In addition, our results differ from a previous study which showed a direct correlation between serious ADRs and age (Lombardi et al., 2018).

Males and females respond differently to drugs, because of differences in anatomic and physiological characteristics. In fact, a systematic review showed a higher risk of ADRs in female children (Smyth et al., 2012). However, a multicenter cohort study involving pediatric patients admitted because of ADRs in hospitals from five countries, did not show substantial differences by gender (Rashed et al., 2012); finally an overlapping number of ADRs in both males and females has been reported in other studies (Aagaard et al., 2010; Aldea et al., 2012). In our study, no difference by gender was observed between patients affected by serious or not serious ADRs.

The Naranjo algorithm is used for the causality assessment of ADRs, but it has some limits, such as the re-administration of the suspected drug or the administration of the placebo. In addition, the validity of this tool has not been demonstrated and fully evaluated in pediatric patients. However, because of its simplicity is used to evaluate the causality score even in the pediatric population (Vázquez-Alvarez et al., 2017). In our study, ADRs resulted only “probable” or “possible”, and no significant difference in seriousness and preventability was observed.

Among serious ADRs, only one case of significant disability was observed (preventable hepatotoxicity related to paracetamol). In particular, it concerned a 2-month-old infant affected by remitting hyperpyrexia, treated with paracetamol (125 mg) 4 times, every 5 h. Considering the paracetamol summary of product characteristics (SmPC), this treatment has to be considered inappropriate causing hepatotoxicity and consequently intensive care admission with increased hospital LOS. This evidence underlines the need to implement among pediatricians the adherence to the therapeutic guidelines and to improve a right drug prescribing pattern.

Indeed, prevention is a key factor in the management of ADRs. In our study most not-preventable ADRs were due to vaccines and infants were the group with a higher prevalence of not-preventable ADRs. Considering the National Plan for Vaccine Prevention, this evidence is not surprising because of most vaccines are administered to infants (Signorelli et al., 2018).

Concerning the therapeutic substances involved in ADRs, our results confirm previously reported data (Clavenna et al., 2009; Piovani et al., 2013; Nogueira Guerra et al., 2015; Ferrajolo et al., 2019). In fact, vaccines were the drug class involved in ADRs occurrence, especially in infants. Pneumococcus purified polysaccharides antigen conjugated vaccine was the most associated with ADRs, with a frequency higher than that observed in other studies (Rosafio et al., 2017). Furthermore, our findings are reinforced by data reported from the Sicilian Pharmacovigilance Regional Center in the same study period. Indeed, throughout the 2012–2018 period, the Regional Pharmacovigilance Center received 3,504 reports: 2,895 were due to vaccines and 609 to drugs. Of these reports, 2.2% of vaccines and 9.5% of drugs derived from the present study.

Antibacterial for systemic use, specifically amoxicillin/beta-lactamase inhibitor, were the most common drugs associated with ADRs in children. This result could be explained in light of the evidence that this antibacterial class is the most prescribed therapeutic groups in the general pediatric population (Piovani et al., 2015; Rosli et al., 2017). This finding suggests the evidence of an inappropriate drug prescribing attitude among pediatricians: in fact, amoxicillin/beta-lactamase inhibitor should not be prescribed, being not included by the World Health Organization (WHO) in the “key access” antibiotic list that should be implemented in the real life to prevent the risk of ADRs and to curb the development of antibiotic resistance.

Moreover, in the present study anti-inflammatory drugs and anti-rheumatic products were the drugs most involved in the development of ADRs among adolescents. Furthermore, in agreement with the literature, the most affected organs or systems were skin and subcutaneous tissue followed by the gastrointestinal tract (Priyadharsini et al., 2011). The reason for the higher cutaneous involvement in children ADRs may be ascribed to the peculiar physiology of this age, in fact, neonates and infants have a partially mature and not fully epidermis (Rashed et al., 2012). Thus, the skin is more permeable and vulnerable to chemical and microbial aggressions (Stamatas et al., 2011).

Our study has some limitations and strengths. First, this is a single-center study which involves the pediatric patients admitted to the ED of the University Hospital “G.Martino” of Messina. However, ours results are in agreement with other Italian centers project. Moreover, our reports could be underestimated due to the difficult to achieve patients’ charts and to the lack of information of these data.

ADRs records are important to monitor the safety of pediatric patients, providing additional literature data, currently scarce. Furthermore, this is the first prospective study that evaluates the admissions to pediatric ED related to an ADR for 7 years in Sicily.

In conclusion, our report confirms that ADRs in the paediatric population impose a significant public health concern, even if it may underestimate the real impact because of the of lack of complete clinical data, and it strongly suggests the need of reinforcing active pharmacovigilance to markedly reduce the health and economic burden for the regional Healthcare System.

## Data Availability Statement

The datasets generated and/or analyzed during the current study are not publicly available due to national pharmacovigilance policies established by the Italian Medicines Agency but are available from the corresponding author on reasonable request under a specific authorization. Requests to access the datasets should be directed to the Italian Medicines Agency or varcoraci@unime.it.

## Ethics Statement

The studies involving human participants were reviewed and approved by Ethics Committee of Messina University Hospital (n° prot E22/12; Coordinator Centre). Written informed consent from the participants’ legal guardian/next of kin was not required to participate in this study in accordance with the national legislation and the institutional requirements.

## Author Contributions

FM-I, MV, and DA contributed conception and design of the study. MR and VA performed the statistical analysis. AM, PC, VS, and GP organized the database. CN, FS, VA, and DA wrote original draft preparation. NI and DA wrote review and editing. DA administrated the project. All authors contributed to the article and approved the submitted version.

## Funding

This work has been supported by Departmental funding assigned to DA.

## Conflict of Interest

The authors declare that the research was conducted in the absence of any commercial or financial relationships that could be construed as a potential conflict of interest.
